# Notices

**Published:** 1863-04

**Authors:** 


### NOTICES.

THE AMERICAN TRACT SOCIETY, 28 Cornhill, Boston, and No. 13 Bible House, New-York City, under the efficient management of Mr. J. G. Broughton, has issued HERBERT; or, True Charity, 45 cents. PATIENCE; or, the Sunshine of the Heart. RUTH AND LITTLE JANE; or, Blossoms of Grace. ROSE; or, The Little Comforter. MARY S. PEAKE, of Fortress Monroe. If our subscribers will refer to the bottom of the last page of the January number, they will find another list (with prices) of the admirable publications of this Society, and we cordially advise our friends who come to New-York, and wish to treat their little ones at home with reading which is at once delightful and instructive, to call on Mr. Broughton, at No. 13 Bible House, and examine his list of books.

ALWAYS THANKFUL, is the suggestive title of a discourse delivered in Springfield, Ohio, November 27th, 1862, by Rev. Sylvester F. Scovel, son of the late lamented and loved President of Hanover College, Indiana. Thankful for *all* things individually, and having faith in God that he will bring this nation safely, gloriously through her present trials, ruling and overruling, and shaping all that man does to the highest happiness of his creatures, and the greatest glory of his own great name. It is a delightful subject, handled wisely and well. "Always Thankful!" having faith in God! what a glorious attainment, and possible to all!

"DENTAL JOURNAL FOR THE PEOPLE." Edited by W. W. Allport, D.D.S., Chicago, Illinois. Published monthly, at fifty cents a year. It is an unoccupied field, and one of very high importance, as it seeks to instruct the people as to the proper care of the teeth. Such a journal, well conducted, ought to be taken by every family in the nation, and we hope Dr. Allport will receive the patronage and encouragement which he evidently merits.

AMERICAN MEDICAL ASSOCIATION meets at Chicago, Ill., June 2d, 1863. By order of Committee of Arrangements. N. S. Davis, M.D., Chairman.

NATIONAL ALMANAC AND ANNUAL RECORD. By Geo. W. Childs & Co., 628 and 630 Chestnut street, Philadelphia; also, A. Roman & Co., San Francisco; N. Trübner & Co., 60 Paternoster Row, London; Hector Bossange, Paris, $1. It is the most comprehensively valuable Almanac ever issued in this country; and as an evidence of its appreciation, the publishers are selling a thousand copies weekly. We advise the copy bound in muslin, at $1.25. It will be a work for reference for many years to come. We will send it full bound. post-paid, for five new subscribers to HALL'S JOURNAL OF HEALTH, at one dollar a year. It is filled with valuable statistics, history of the States, officers of the United States and the so-called Confederates; obituaries; census, banks, tariffs, public laws, books published in the United States in 1862, excise tax, post-office department, etc. etc.

